# Supplementary material for: Detailed molecular and epigenetic characterization of the pig IPEC-J2 and chicken SL-29 cell lines
Source: iScience. 2023 Feb 20;26(3):106252. doi: 10.1016/j.isci.2023.106252 (PMC10018572; doi:10.1016/j.isci.2023.106252)
Supplement: Data S2. Complete homer output for identified motifs in Chicken SL-29, related to Tables 5 and 6 — Homer motif analysis results for histone modifications H3K4me1, H3K4me3, H3K27ac, enhancers, and ATAC-seq of chicken SL-29 cell line. Parameters for possible false positives is as mentioned earlier for S5. [file mmc3.zip › Data_S2/S6/Chicken_SL_29/peak_files_enhancers/homerResults/motif1.similar.html]

motif1

## Information for motif1

A
G
C
T
A
C
T
G
C
G
T
A
A
T
C
G
A
C
G
T
G
T
A
C
C
G
T
A
A
G
C
T
  
Reverse Opposite:  

T
C
G
A
A
C
G
T
A
C
T
G
C
G
T
A
T
A
G
C
A
C
G
T
G
T
A
C
C
T
G
A
  

|  |  |
| --- | --- |
| p-value: | 1e-525 |
| log p-value: | -1.210e+03 |
| Information Content per bp: | 1.860 |
| Number of Target Sequences with motif | 3182.0 |
| Percentage of Target Sequences with motif | 17.19% |
| Number of Background Sequences with motif | 2076.7 |
| Percentage of Background Sequences with motif | 6.59% |
| Average Position of motif in Targets | 146.1 +/- 80.4bp |
| Average Position of motif in Background | 150.4 +/- 86.5bp |
| Strand Bias (log2 ratio + to - strand density) | 0.0 |
| Multiplicity (# of sites on avg that occur together) | 1.12 |
| Motif File: | file (matrix) reverse opposite |

### Similar de novo motifs found

|  |  |  |  |  |  |  |  |
| --- | --- | --- | --- | --- | --- | --- | --- |
| Rank | Match Score | Redundant Motif | P-value | log P-value | % of Targets | % of Background | Motif file |
| 1 | 0.955 | A C G T A C T G C G T A A T G C A C G T A G T C C G T A A C G T G T A C T C A G | 1e-505 | -1164.088784 | 14.78% | 5.25% | motif file (matrix) |
| 2 | 0.962 | G C T A C T G A T C A G T G C A C G A T T G C A C G A T A C T G C G T A A T C G A G C T A T G C C G T A | 1e-459 | -1057.065285 | 18.21% | 7.77% | motif file (matrix) |
| 3 | 0.952 | A G C T A C T G T G C A A T C G C A G T T G A C T C G A A G C T | 1e-428 | -987.196697 | 26.05% | 13.71% | motif file (matrix) |
| 4 | 0.890 | C T G A A C G T A C T G C G T A C G T A A C G T A G T C C G T A A G T C T G C A | 1e-106 | -245.219331 | 4.71% | 2.05% | motif file (matrix) |
| 5 | 0.682 | T A G C T A G C C A G T A G C T G T C A C T A G A C G T G T A C | 1e-76 | -175.657759 | 26.13% | 20.47% | motif file (matrix) |
| 6 | 0.783 | A C G T T C G A C G A T A G C T T C G A T A C G C A G T T G A C T G C A A G C T | 1e-72 | -167.702933 | 5.47% | 2.96% | motif file (matrix) |
| 7 | 0.653 | C G A T A G C T C G T A A T C G C A G T G T A C C G T A A G C T A G C T A C G T A G T C G A T C G A T C | 1e-31 | -71.893417 | 0.14% | 0.00% | motif file (matrix) |
| 8 | 0.644 | C T A G T C G A A C G T A C T G C G T A A T G C C G A T C T G A G T C A A C G T A C T G T G A C G A T C | 1e-30 | -70.993321 | 0.16% | 0.01% | motif file (matrix) |
| 9 | 0.632 | A C T G G C A T C T G A C A G T A C T G C G T A G T A C G C A T C T G A C G T A C T A G T G A C G C T A | 1e-27 | -63.414826 | 0.22% | 0.02% | motif file (matrix) |
| 10 | 0.724 | G T A C A C G T A C T G A C G T A C T G A G C T G T A C C G T A | 1e-20 | -46.589101 | 10.54% | 8.56% | motif file (matrix) |
